# Lower heart rate variability is associated with worse memory function: The Maastricht Study

**DOI:** 10.1177/13872877251389985

**Published:** 2025-10-29

**Authors:** Chidera Okoro, Tan Lai Zhou, Carla JH van der Kallen, Sebastian Kohler, Miranda T Schram, Abraham A Kroon

**Affiliations:** 1 CARIM School for Cardiovascular Diseases, Maastricht University (UM), Maastricht, the Netherlands; 2 Department of Internal Medicine, Maastricht University Medical Center^+^, Maastricht, the Netherlands; 3 MHeNS Mental Health and Neuroscience Research Institute, Maastricht University (UM), Maastricht, the Netherlands; 4 Department of Psychiatry and Neuropsychology, Alzheimer Centre Limburg, Maastricht University Medical Center+, Maastricht, the Netherlands; 5 Heart and Vascular Centre, Maastricht University Medical Center+, Maastricht, the Netherlands; 6 Department of Epidemiology, Erasmus MC, Rotterdam, the Netherlands

**Keywords:** Alzheimer's disease, autonomic nervous system, heart rate, memory disorders

## Abstract

**Background:**

With increasing life expectancy, more individuals will face age-related cognitive difficulties, including Alzheimer's disease. It is, therefore, important to identify early, potentially modifiable risk factors for cognitive impairment. Lower heart rate variability (HRV), reflecting worse cardiac autonomic function, may be one such factor, though its link to cognition remains unclear.

**Objective:**

This study investigates whether lower HRV is associated with worse cognitive performance.

**Methods:**

We used population-based cross-sectional data from The Maastricht Study (N = 9187 participants, mean age 59.5 years, 50.2% women, 21% with type 2 diabetes) to investigate the associations of 24-h HRV with memory function, executive function, and information processing speed. Linear regression analyses adjusted for potential cardiovascular confounders were conducted, and we tested for interactions by sex, age, and glucose metabolism status (prediabetes and type 2 diabetes versus normal glucose metabolism).

**Results:**

After adjusting for covariates, both lower time and frequency domain HRV (per 1 standard deviation [SD]) were associated with worse memory function (−0.04 SD [95%CI −0.06; −0.02] and −0.02 SD [95%CI −0.04; −0.00], respectively), equivalent to aging by 1–1.5 years. HRV was not significantly associated with executive function (−0.00 SD [95%CI −0.02; 0.02] and −0.02 SD [95%CI −0.04; 0.00]) or information processing speed (0.01 SD [95%CI −0.01; 0.03] and −0.00 SD [95%CI −0.02; 0.02]). No interactions with sex, age, or glucose metabolism status were found.

**Conclusions:**

Lower HRV was associated with worse memory function in mid- to late-life individuals, independent of cardiovascular risk factors.

## Introduction

The global population is aging rapidly. As a consequence, more individuals will face cognitive difficulties, including dementia,^1^ which currently affects over 55 million people worldwide and its prevalence is estimated to rise to 80 million by 2030 according to the World Health Organization.^2^ It is crucial to identify and treat, potentially modifiable risk factors of cognitive impairment in order to delay the disease progression as effectively as possible. One biological system that has been increasingly recognized as relevant to cognitive health is the autonomic nervous system, which regulates cardiovascular function, cerebral blood flow, and inflammatory responses. Cardiac autonomic function, commonly assessed by heart rate variability (HRV),^3^ may influence cognitive performance.^4,5^ Reduced HRV reflects impaired autonomic balance, which may contribute to cerebral hypoperfusion^6^ and increased neuroinflammation.^7^ These pathways may ultimately result in cerebral (micro)vascular damage that underlies cognitive decline.^8^ Therefore, we investigated whether lower HRV is associated with worse cognitive performance.

Past studies, both small^9–14^ and large,^15–18^ have yielded conflicting results regarding the association between HRV and cognitive performance. This may be due to the fact that previous studies predominantly focused on short-term HRV recordings (<2 h), used only a single measure of HRV (e.g., standard deviation only or frequency domain variables only),^11,16^ assessed only global cognitive performance (e.g., the Mini-Mental State Exam),^12^ tested only one domain of cognitive performance,^11,17^ or focused on specific populations (e.g., old to very old individuals (>70 years old)).^12,13,16^

In view of the above, we investigated the associations of both time and frequency domains of 24-h HRV measurements with global cognitive performance consisting of three domains of cognitive performance (i.e., memory function, information processing speed and executive function) in mid- to late-life (40- to 75-year-old) individuals from the population-based Maastricht Study.

## Methods

### Study population and design

We used data from The Maastricht Study, a prospectively designed, population-based observational cohort study. The rationale and methodology have been described previously.^19^ In brief, the study focuses on the etiology, pathophysiology, complications, and comorbidities of type 2 diabetes mellitus and is characterized by an extensive phenotyping approach. All individuals aged 40–75 years who lived in the southern part of the Netherlands were eligible for participation.^19^ The present report includes cross-sectional data of 9187 participants who were included in the baseline survey between November 2010 and October 2020. The examinations of each participant were performed within a time window of three months*.* The study has been approved by the institutional medical ethical committee (NL31329.068.10) and the Minister of Health, Welfare, and Sports of the Netherlands (Permit 131088-105234-PG). All participants gave written informed consent.^19^

### Assessment of heart rate variability

For the extended methodology, we refer to the Supplemental Methods section.

As described previously,^20^ ECGs were recorded using a 12-lead Holter system (Fysiologic ECG Services, Amsterdam, the Netherlands) over a 24-h period, during which participants maintained their usual activities but avoided bathing. Recordings were analyzed with proprietary Holter Analysis Software and validated by manual inspection afterward. Time domain measures included SDNN (SD of all normal-to-normal intervals), SDANN (SD of averages in 5-min segments), RMSSD (square root of the mean of squared differences between adjacent intervals), SDNN index (mean of SDs of 5-min segments), and pNN50 (percentage of adjacent intervals differing by >50 ms). Frequency domain measures were derived using Fast Fourier Transform, including total power (TP), ultralow frequency (ULF ≤0.003 Hz), very-low frequency (VLF 0.003–0.04 Hz), low frequency (LF 0.04–0.15 Hz), and high frequency (HF 0.15–0.4 Hz). Composite z-scores were calculated for time (SDNN, RMSSD, SDANN, SDNN index, pNN50) and frequency domain measures (TP, ULF, VLF, LF, HF), excluding SDSD, NN50, and LF/HF as they were derived from variables already included in the overall score. Individuals with pacemakers and atrial fibrillation were excluded as HRV will be inaccurate due to these conditions (n = 63).

### Assessment of cognitive performance

Cognitive performance was assessed by a brief 30-min neuropsychological test battery.^21^ Scores were standardized and grouped into three domains: memory function, information processing speed, and executive function. Episodic memory function was evaluated using the Verbal Learning Test,^22^ by averaging immediate and delayed recall scores. The composite score for information processing speed was derived from the averaging the standardized scores of the Stroop Color-Word Test Part I and II,^23^ the Concept Shifting Test Part A and B,^24^ and the Letter-Digit Substitution Test.^25^ Executive function was assessed by averaging standardized scores of the Stroop Color-Word Test Part III and the Concept Shifting Test Part C. Scores were log-transformed or inverted as needed, then standardized to a mean of zero and SD of 1. Global cognitive performance was represented by a composite score derived from the three cognitive domains.

### Covariates

As previously described,^21^ we used fasting plasma glucose and 2-h postload glucose to assess glucose metabolism status according to the World Health Organization 2006 criteria as normal glucose metabolism, prediabetes, type 2 diabetes, type 1 diabetes, or other types of diabetes. Alcohol consumption, smoking status and educational level were assessed by questionnaire. Alcohol consumption was defined as non-consumer, low-consumer (≤7 alcoholic drinks/week for women; ≤14 alcoholic drinks/week for men), and high-consumer (>7 alcoholic drinks/week for women; >14 alcohol drinks/week for men). Smoking status was categorized into never, former, and current smoker. Educational level was classified into 3 groups: low (none, primary or lower vocational education only), medium (intermediate general secondary, intermediate vocational or higher general secondary education), and high (higher vocational education or university level of education).^21^; Information on the use of anti-depressive, anxiolytic, sleep-modifying, lipid-modifying and antihypertensive medications was collected during an interview. Physical activity was assessed using an accelerometer.^26^ Waist circumference and office blood pressure were assessed as part of a physical examination; cholesterol levels were determined in fasting blood samples; kidney variables were assessed as estimated glomerular filtration rate and albuminuria.^21,27^ A validated food frequency questionnaire was used to assess the Dutch Healthy Diet score.^28^ A current major depressive episode was assessed by the Mini-International Neuropsychiatric Interview.^29^

### Statistical analyses

Continuous variables are reported as mean (SD) or median [25th and 75th percentiles] depending on normality, while categorical variables are expressed as percentages.

We used multivariable linear regression to investigate associations of composite HRV measures (time and frequency domain) with cognitive domains (memory, executive function, information processing speed) and global cognitive performance. Results are presented as standardized regression coefficients (β) with 95% confidence intervals (CIs).

Model 1 shows crude results. Model 2 was adjusted for age, sex, education and glucose metabolism status. We chose these variables because they are key potential confounders and because of the oversampling of participants with type 2 diabetes. Model 3 was additionally adjusted for waist circumference, alcohol consumption, smoking status, total cholesterol-to-HDL cholesterol ratio, office systolic blood pressure, use of lipid-modifying and anti-hypertensive medications. We adjusted for these covariates in model 3 as they have previously been associated with lower cognitive performance and could affect HRV.^30^

### Additional analyses

Additional analyses included repeating the main analysis with individual HRV measures, replacing certain covariates (e.g., waist circumference with BMI, glucose metabolism status with continuous measures of glycemia, systolic blood pressure with diastolic blood pressure; and educational level with income), and adjusting for physical activity and diet score. We also tested associations excluding individuals using antihypertensive medications and adjusted for kidney function, depression, and medication use. Interaction analyses were performed by sex, age, and glucose metabolism status to evaluate whether associations differed by gender, age, or glucose metabolism (type 2 diabetes, prediabetes, or normal glucose metabolism).

For the interaction analyses involving glucose metabolism status, we excluded participants with other types of diabetes (n = 35).

All analyses were performed with SPSS version 25.0 (IBM SPSS, IBM Corp, Armonk, NY, USA). For all analyses, a two-sided p-value <0.05 was considered statistically significant.

## Results

### Selection and characteristics of the study population

**Figure 1.**
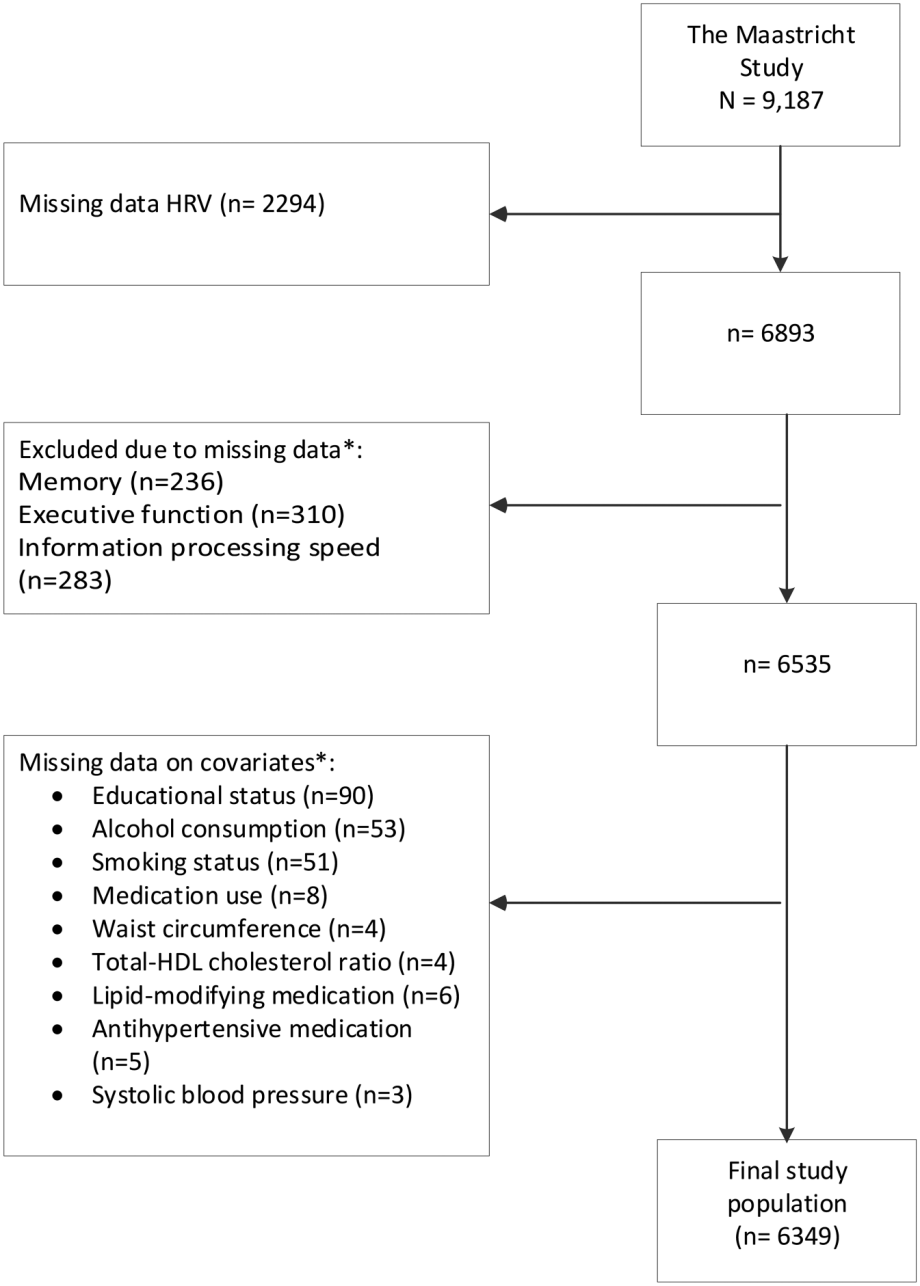
Flowchart delineating the derivation of the final study population. * not mutually exclusive. HRV: heart rate variability; HDL: high density lipoprotein.

Complete data on HRV, covariates, and cognitive performance were available for 6349 participants (Figure 1). Table 1 shows general characteristics of the population according to tertiles of memory function. Participants in the lowest tertile of memory function were older, more often male, less educated, and had worse cardiovascular profiles. Those excluded due to missing data had a worse risk profile, with higher waist circumference and poorer cognitive performance compared to those included (Supplemental Table 1).

**Table 1.** General study population characteristics according to tertiles of memory function with complete data on heart rate variability.

Characteristic	Tertiles of Memory function			
	Total study group (N = 6349)	Tertile 1 (low) (N = 2111)	Tertile 2 (middle) (N = 2106)	Tertile 3 (high) (N = 2132)
**Demographic characteristics**				
Age (y)	59.35 ± 8.66	62.63 ± 8.00	59.45 ± 8.28	56.01 ± 8.40
Female, No. (%)	3213 (50.6)	678 (32.1)	1038 (49.3)	1497 (70.2)
Educational level				
Low	2024 (31.9)	911 (43.2)	709 (33.7)	404 (18.9)
Middle	1769 (27.9)	575 (27.2)	577 (27.4)	617 (28.9)
High	2556 (40.3)	625 (29.6)	820 (38.9)	1111 (52.1)
**Cardiovascular risk factors**				
Glucose metabolism status				
Normal glucose metabolism	4015 (63.2)	1106 (52.4)	1345 (63.9)	1564 (73.4)
Prediabetes	949 (14.9)	382 (18.1)	299 (14.2)	268 (12.6)
Type 2 diabetes	1350 (21.3)	611 (28.9)	452 (21.5)	287 (13.5)
Type 1 and other type of diabetes	35 (0.6)	12 (0.6)	10 (0.5)	13 (0.6)
Waist circumference (cm)	94.64 ± 13.48	98.35 ± 13.19	95.00 ± 13.21	90.61 ± 12.91
Total/HDL cholesterol ratio	3.63 ± 1.19	3.74 ± 1.25	3.66 ± 1.19	3.49 ± 1.10
Systolic blood pressure (mm Hg)	133.21 ± 17.89	137.27 ± 17.92	133.75 ± 17.38	128.65 ± 17.32
**Medications**				
Use of lipid-modifying medication (yes versus no)	1825 (28.7)	791 (37.5)	618 (29.3)	416 (19.5)
Use of blood pressure medications (yes versus no)	2218 (34.9)	943 (44.7)	740 (35.1)	535 (25.1)
**Lifestyle factors**				
Alcohol consumption				
None	1132 (17.8)	401 (19.0)	382 (18.1)	349 (16.4)
Low (women<=7,men<=14)	3734 (58.8)	1261 (59.7)	1244 (59.1)	1229 (57.6)
High (women > 7,men > 14)	1483 (23.4)	449 (21.3)	480 (22.8)	554 (26.0)
Smoking status				
Never	2406 (37.9)	710 (33.6)	800 (38.0)	896 (42.0)
Former	3136 (49.4)	1102 (52.2)	1038 (49.3)	996 (46.7)
Current	807 (12.7)	299 (14.2)	268 (12.7)	240 (11.3)
**Heart rate variability**				
**Time Domain**				
Composite score, SD	0.00 ± 1.00	−0.08 ± 0.91	−0.02 ± 0.95	0.10 ± 1.11
SDNN, ms	142.08 ± 74.70	138.83 ± 76.34	141.80 ± 71.40	145.57 ± 76.11
SDANN, ms	134.16 ± 96.18	129.35 ± 90.25	133.66 ± 93.32	139.42 ± 104.10
RMSSD, ms	43.51 ± 67.99	39.58 ± 57.76	41.11 ± 65.38	49.79 ± 78.63
SDNN index, ms	79.06 ± 115.36	70.45 ± 96.69	75.91 ± 108.531	90.70 ± 136.12
pNN50, %	6.75 (2.90–14.05)	5.94 (2.49–12.32)	6.52 (2.81–13.90)	7.74 (3.54–15.98)
**Frequency Domain**				
Composite score, SD	0.00 ± 1.00	−0.09 ± 0.98	0.00 ± 1.01	0.09 ± 1.00
TP, ms^2^	13,067 ± 7439	12,462 ± 7223	13,117 ± 7614	13,617 ± 7435
ULF, ms^2^	11,182 ± 6653	10,667 ± 6448	11,233 ± 6822	11,656 ± 6649
VLF, ms^2^	1283 ± 878	1237 ± 887	1287 ± 899	1323 ± 845
LF, ms^2^	471 ± 390	440 ± 392	471 ± 383	501 ± 392
HF, ms^2^	92.82 (51.58– 169.37)	80.3 (45.65– 148.88)	90.02 (51.27– 167.17)	105.44 (60.24– 192.70)
**Cognitive Performance**				
Memory Function	0.04 ± 0.94	−1.01 ± 0.50	0.05 ± 0.24	1.07 ± 0.41
Information Processing Speed	0.03 ± 0.77	−0.36 ± 0.76	0.05 ± 0.69	0.41 ± 0.68
Executive function	0.03 ± 0.80	−0.26 ± 0.84	0.04 ± 0.74	0.31 ± 0.70

Data are presented as mean ± standard deviation, median [interquartile range] or number (%).

SD: standard deviation; HDL: high-density lipoprotein.

### Association with global cognitive performance

**Figure 2.**
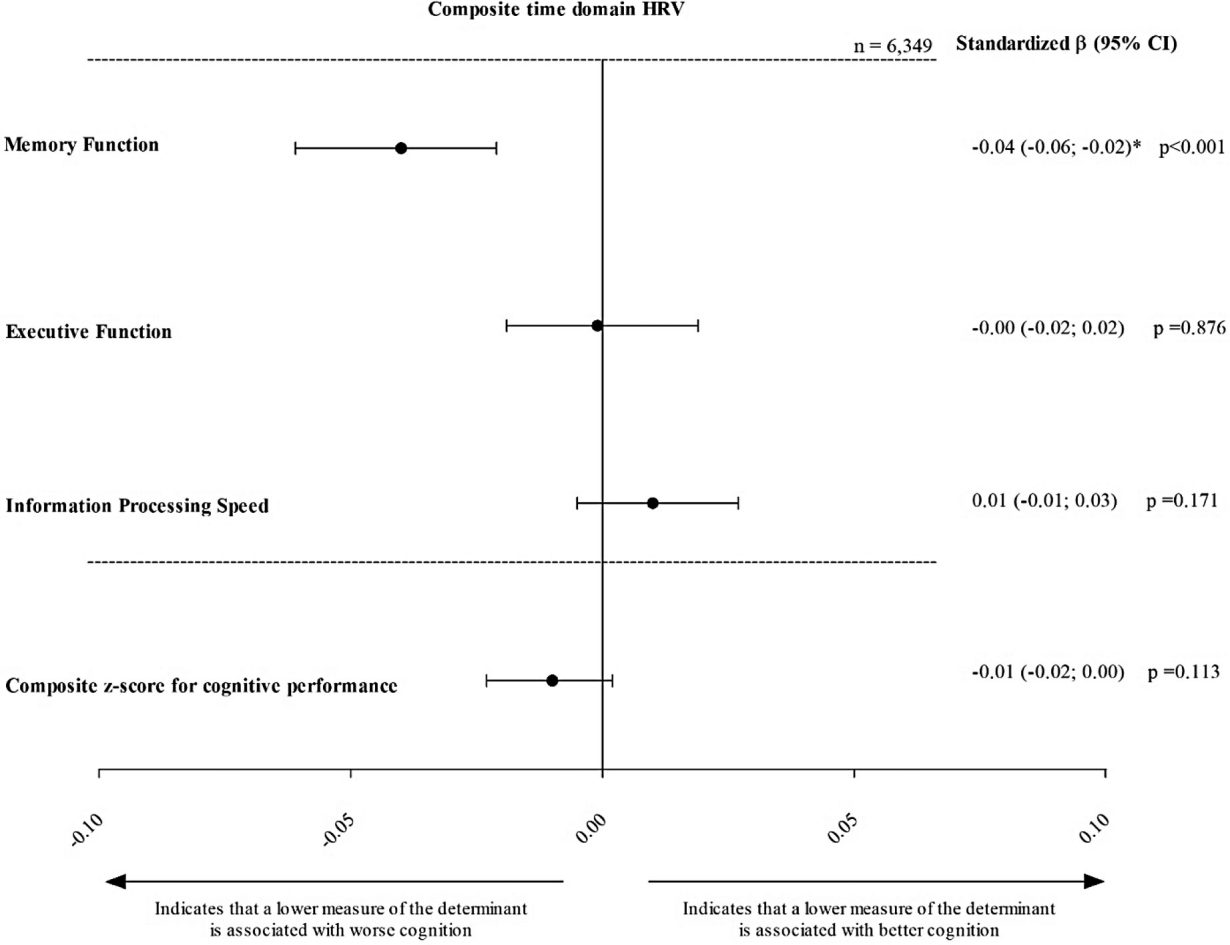
Association of time domain composite z-score HRV with memory function, executive function and information processing speed. Standardized regression coefficients (stβ) represent the differences in global cognitive performance, memory function, executive function and information processing speed in SD, for every 1 standard deviation (SD) lower time domain composite z-score HRV. Results are adjusted for age, sex, glucose metabolism status, educational level, waist, alcohol consumption status, smoking status, total cholesterol-to-HDL cholesterol ratio, use of lipid-modifying medication, use of anti-hypertensive medication and office systolic blood pressure. *p < 0.05. Time domain z-score combines SDNN, SDANN, RMSSD, SDNN index and pNN50. Stβ: standardized beta; CI: confidence interval; SD: standard deviation.

**Figure 3.**
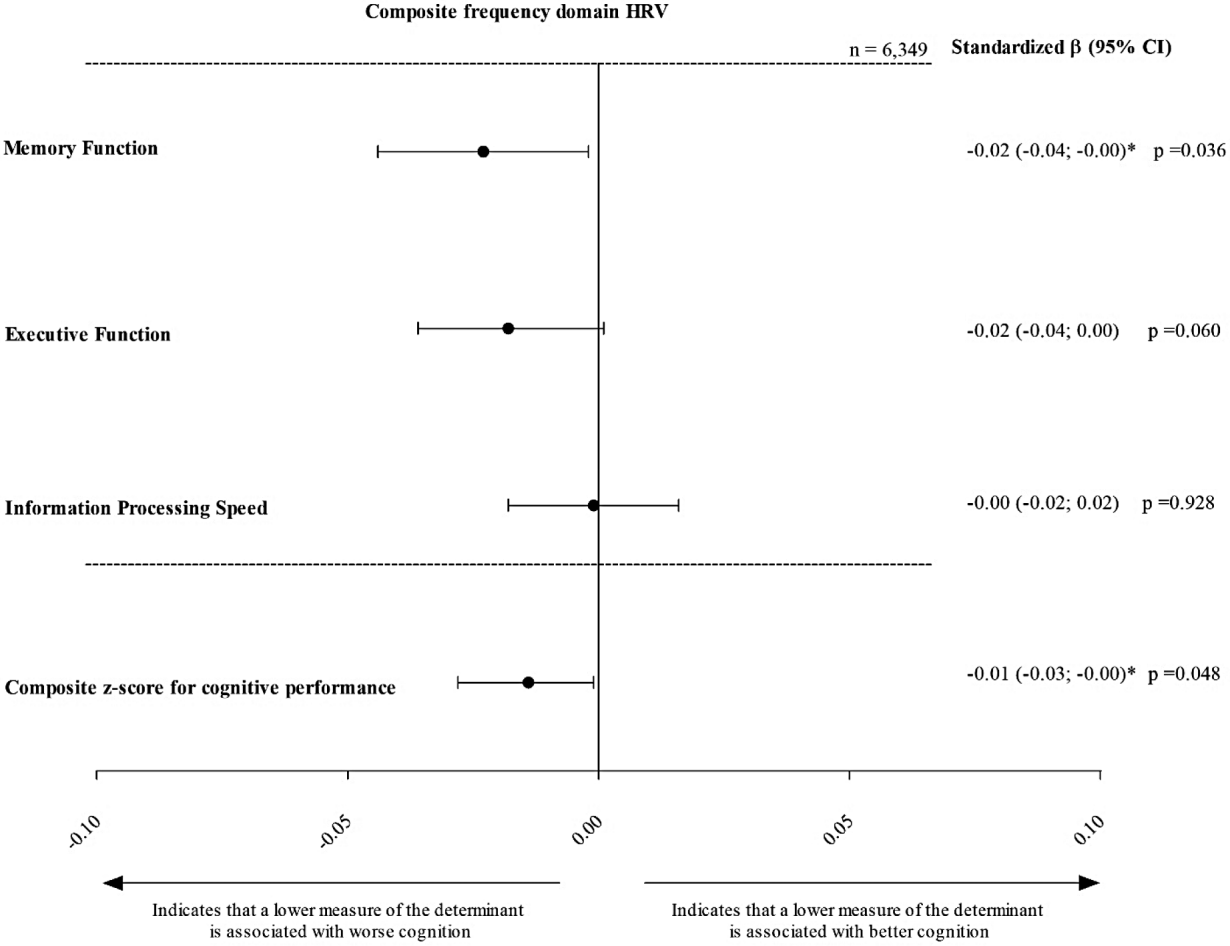
Association of frequency domain composite z-score HRV with memory function, executive function and information processing speed. Standardized regression coefficients (stβ) represent the differences in global cognitive performance, memory function, executive function and information processing speed in SD, for every 1 standard deviation (SD) lower frequency domain composite z-score HRV. Results are adjusted for age, sex, glucose metabolism status, educational level, waist, alcohol consumption status, smoking status, total cholesterol-to-HDL cholesterol ratio, use of lipid-modifying medication, use of anti-hypertensive medication and office systolic blood pressure. * denotes p < 0.05. Frequency domain z-score combines TP, ULF, VLF, LF, and HF. Stβ: standardized beta; CI: confidence interval; SD: standard deviation.

After adjustment for the covariates of model 3, composite time domain HRV was not statistically significantly associated with global cognitive performance (stβ [95% CI], −0.01[ 0.00; −0.02]). However, lower composite frequency domain HRV was statistically significantly associated with lower global cognitive performance (−0.01 [ −0.00; −0.03]; Figures 2 and 3, Supplemental Table 2).

### Association with cognitive domains

For memory function, lower composite time and frequency domain HRV were both significantly associated with lower memory function (−0.04 [−0.06; −0.02], p < 0.001 and −0.02 [−0.04; −0.00], p = 0.036, respectively). To contextualize the magnitude of these associations, we estimated the mean change in memory z-scores attributable to aging within our sample, which was approximately −0.027 SD per year. Accordingly, the observed association between lower HRV and memory function (–0.04 SD) corresponds to the effect size typically seen with approximately 1.5 years of aging (i.e., −0.04 / −0.027 = approx. 1.48 years).^31,32^ No significant associations were found for executive function or information processing speed (Supplemental Table 2, Figures 2 and 3).

### Additional analysis

When individual time domain HRV components were analyzed, SDANN (−0.03 [−0.005 to −0.045], p = 0.016), RMSSD (−0.03 [−0.013 to −0.053], p = 0.001), SDNN index (−0.04 [−0.022 to −0.062], p < 0.001), and pNN50 (−0.04 [−0.020 to −0.060], p < 0.001), except SDNN (−0.01 [0.007 to −0.033], p = 0.169) showed significant associations with memory function, but none with executive function. Only RMSSD (0.02 [0.033 to 0.001], p = 0.038) showed a significant association with information processing speed. For frequency domain HRV, TP (−0.02 [−0.001 to −0.043], p = 0.038), ULF (−0.02 [−0.000 to −0.042], p = 0.049), and VLF (−0.02 [−0.002 to −0.045], p = 0.031) were associated with memory function, VLF (−0.03 [−0.011 to −0.049], p = 0.002) and LF (−0.02 [−0.000 to −0.038], p = 0.048) with executive function, and ULF (−0.02 [−0.000 to −0.033], p = 0.044) and HF (0.02 [0.035 to 0.003], p = 0.018) with information processing speed (Supplemental Tables 3 and 4).

Associations did not materially change after replacing waist circumference with BMI, glucose metabolism status with continuous glycemia measures, or after adjusting for estimated glomerular filtration rate, depressive symptoms, medication use, and other factors. However, associations between time domain HRV and memory function attenuated after adjusting for physical activity and diet due to missing data (Supplemental Tables 5–7).

Age, sex, and glucose metabolism did not modify the associations between HRV and cognitive performance (Supplemental Table 8).

## Discussion

The main finding of this study is that both lower time and frequency domain HRV measures are independently associated with worse memory function, but not with information processing speed, or executive function in a large cohort of mid- to late-life community-dwelling individuals. The strength of this association was, for each SD lower HRV, equivalent to approximately 1–1.5 years of older age with regard to memory function. In addition, we observed an association between lower frequency domain HRV and worse global cognitive performance, which is likely mostly driven by a lower memory function.

Our study adds novel findings to the existing literature, as it was performed in a large cohort of community-dwelling individuals in mid- to late-life, applied an extensive cognitive test battery, and utilized 24-h HRV measurements. Previous studies, in contrast, were relatively small,^10–13^ focused on specific study populations, such as white-collar workers,^15^ only females,^12^ older to very old individuals,^12,13,16^ middle-aged male twins,^9^ assessed only global cognitive performance, such as the Mini-Mental State Exam^12^ and predominantly used short-term HRV measurements of 10 s,^16,33,34^ 5 min,^10,11,13–15,18^ 10 min,^17^ 2 h^12^ and heart rate fragmentation (HRF) assessed during sleep.^35^ Of note, the 24-h HRV measurement is more accurate than the short-term, providing detailed physiological information, capturing circadian autonomic fluctuations, offering greater sensitivity and reproducibility^36^ and potentially having the highest prognostic value.^37^

Interestingly, we only observed a significant association between lower HRV and worse memory function. Several mechanisms may explain how HRV influences cognitive performance and preferentially memory function. First, the balance between cardiac sympathetic and parasympathetic activity helps to maintain normal blood pressure and ensures adequate cerebral blood flow.^38^ When HRV is impaired, cerebral blood flow may become inadequate via various hemodynamic changes, to which the brain is especially vulnerable due to the relatively low impedance of brain vasculature. This may lead to cerebral ischemia and if this happens in the temporal lobes, where the hippocampus is located (i.e., the brain region for memory consolidation), memory function is impaired. Indeed, previous studies show that the temporal lobe, especially the medial region, is more vulnerable to cerebral ischemia.^39^ Second, the observed association between lower HRV and worse memory function may involve the fornix,^9,14^ the main outflow tract of the hippocampus which has been associated with autonomic control.^40^ In addition, specific neurons in the cardiac autonomic control center of the brain was shown to relay signals through multiple pathways to the hippocampus.^40^ We therefore hypothesize that poor cardiac autonomic regulation, indicated by lower HRV values, may impair hippocampal function and hinder memory processes, leading to worse memory function as observed in our study. Furthermore, when examining individual HRV measures, we found that for time domain measures, all individual measures were associated with worse memory function except for SDNN. SDNN reflects total variability of the heart rate, which may be less specific as how cognitive impairment is influenced by the autonomic nervous system. For frequency domain measures, ultra-low frequency (ULF), and very low frequency (VLF) were significantly associated with memory function. ULF is primarily influenced by circadian rhythms^41^ which has a pronounced influence on memory function. VLF has been associated with parasympathetic nervous system (PNS) activity, which has been linked to cognitive performance and memory.^42^ Additionally, low VLF has been linked with increased chronic inflammation.^43^ This may result from impaired cholinergic anti-inflammatory function of the cardiac autonomic nervous system, which may lead to cognitive impairment and dementia.^8^

Our findings did not show a clear association between lower HRV and worse executive functioning. This provides a different perspective on the Neurovisceral Integration Model, which proposes that the prefrontal cortex, next to executive functioning, also regulates heart rate and thus HRV.^4^ This could further expand the Neurovisceral Integration Model, as our findings suggest that the association between lower HRV and worse memory function may be independent of executive functioning, for example via subcortical areas or brain regions that regulate emotion. More in-depth investigation on how HRV and memory are connected other than via prefrontal pathways within the Neurovisceral Integration Model is needed. Another explanation as why we did not find any significant associations between HRV and executive function or information processing speed may be due to the fact that lower HRV is an especially important risk factor for older individuals.^44^ Previous studies have shown that lower HRV is mainly linked to decline in executive function and information processing speed in those aged over 75 years old,^13,16^ whereas the average age of our study population was 59 years.

Our findings may suggest that improving the balance in cardiac autonomic function may be protective against cognitive impairment, particularly in preserving memory function. This opens the possibility for interventions such as exercise therapy^45^ aimed at improving cardiac autonomic regulation as a preventive measure for cognitive decline. This may be especially important in those with lower HRV, such as patients with (pre)diabetes,^20^ hypertension^46^ and depression.^47^ However, interventional studies are needed to confirm these associations and to explore potential therapeutic strategies that could improve HRV and thus prevent cognitive impairment.

A major strength of the present study is the use of 24-h HRV measurement, a more accurate assessment of cardiac autonomic function compared to short-term measurement.^48^ Additionally, our study benefited from a large and well-characterized participant cohort, which allowed us to adjust for a large series of confounders. For instance, cardiovascular risk factors such as hypertension,^49,50^ obesity^51,52^ and type 2 diabetes^53,54^ have been linked with both HRV and cognitive performance. However, our findings showed that HRV was associated with memory function independent of these cardiovascular risk factors.

Our study had some limitations. First, the cross-sectional nature of our study implies that causal inference should be made with caution as the reverse association may also hold true. For instance, impaired memory function might increase stress and anxiety levels, which in turn could negatively influence cardiac autonomic nervous system regulation. Additionally, damage to the fornix could lead to lower HRV, indicating that lower HRV might be an indicator of medial temporal lobe neurodegeneration. Second, the possibility of residual confounding factors cannot be entirely ruled out, such as family history of hypertension^55^ and genetic predisposition, for, e.g., catechol-O-methyltransferase (COMT), a dopamine gene that has been linked to both cognitive function^56^ and autonomic function.^57^ Third, our results may be underestimated, as the individuals excluded from this analysis due to missing data had a worse risk profile compared to those who were included. Fourth, some variables had a significant amount of missing data, such as dietary habits and physical activity (n = 1453). The results from these additional analyses should be interpreted with caution as this may have introduced selection bias (Supplemental Table 5). Fifth, although we performed multiple comparisons, we did not adjust for multiple testing, as we had a plausible mechanisms for a single hypothesis,^58^ and aimed to reduce the amount of multiple comparisons by composing a composite score of various HRV measures.

### Conclusion

Our study shows an association between lower HRV and worse memory function among mid- to late-life community-dwelling individuals, independent of cardiovascular risk factors. Further investigations of the underlying mechanisms using brain imaging studies are imperative to gain a deeper understanding of our findings. In addition, intervention trials improving autonomic balance are needed to assess whether this is indeed protective of future cognitive impairment.

## Supplemental Material

sj-docx-1-alz-10.1177_13872877251389985 - Supplemental material for Lower heart rate variability is associated with worse memory function: The Maastricht Study Supplemental material, sj-docx-1-alz-10.1177_13872877251389985 for Lower heart rate variability is associated with worse memory function: The Maastricht Study by Chidera Okoro, Tan Lai Zhou, Carla JH van der Kallen, Sebastian Kohler, Miranda T Schram and Abraham A Kroon in Journal of Alzheimer's Disease
