# Supplementary material for: Lower heart rate variability is associated with worse memory function: The Maastricht Study
Source: J Alzheimers Dis. 2025 Oct 29;108(4):1738–48. doi: 10.1177/13872877251389985 (PMC12664935; doi:10.1177/13872877251389985)
Supplement: sj-docx-1-alz-10.1177_13872877251389985 - Supplemental material for Lower heart rate variability is associated with worse memory function: The Maastricht Study [file sj-docx-1-alz-10.1177_13872877251389985.docx]

**Supplemental Material**

**Lower heart rate variability is associated with worse memory function: The Maastricht Study**

**Supplemental Methods**

*Assessment of heart rate variability*

All ECGs were recorded by use of a 12-lead Holter system (Fysiologic ECG Services, Amsterdam, the Netherlands) over a 24-h period. During recording time, participants were asked to follow their normal daily activities, except that they were asked not to take a shower or a bath. Recordings were analyzed with proprietary Holter Analysis Software at Fysiologic ECG Services with an algorithm that excluded nonsinus cardiac cycles (e.g., artifacts and premature/ectopic beats), validated by manual inspection afterward. The software from Fysiologic provided the intervals between the individual R waves of sinus beats (i.e., interbeat intervals [IBIs], in milliseconds). From the obtained IBIs, HRV was calculated by use of the publicly available free GNU Octave software^1^, according to the standard time and frequency domain measures defined by the 1996 Task Force document on HRV^2^ and recently updated recommendations^3^. The minimum duration of the recording for HRV analysis was 18 h after exclusion of nonsinus cardiac cycles. As for time domain measures, we calculated the SD of all normal-to-normal (NN) intervals (SDNN, in milliseconds); the SD of the averages of NN intervals in all 5-min segments of the entire recording (SDANN, in milliseconds); the square root of the mean of the sum of squares of differences between adjacent NN intervals (RMSSD, in ms); the mean of the SDs of all NN intervals for all 5-min segments of the entire recording (SDNN index, in milliseconds); the SDs of differences between adjacent NN intervals (SDSD, in milliseconds); the number of pairs of adjacent NN intervals differing by >50 ms in the entire recording (NN50 count); and the NN50 count divided by the total number of all NN intervals (pNN50, a percentage). As for frequency domain measures, we used the Fast Fourier Transform to calculate the variance of all NN intervals ≤0.4 Hz (total power [TP], in milliseconds squared); power in the ultralow-frequency range (ULF, in milliseconds squared) (≤0.003 Hz); power in the very-low-frequency range (VLF, in milliseconds squared) (0.003–0.04 Hz); power in the low-frequency range (LF, in milliseconds squared) (0.04–0.15 Hz); power in the high-frequency range (HF, in milliseconds squared) (0.15–0.4 Hz). Individual *z*-scores were calculated for the time and frequency domain measures and combined in an overall time domain variable (SDNN, RMSSD, SDANN, SDNN index, pNN50) and an overall frequency domain variable (TP, ULF, VLF, LF, HF). SDSD and NN50 were excluded from the *z*-score calculation due to the fact that these variables were derived from variables already included in the overall score (i.e., SDNN and SDANN). Individuals with pacemakers and atrial fibrillation were excluded as HRV will be inaccurate due to these conditions (n=63).

*Assessment of cognitive performance*

The composite memory score was derived from the Verbal Learning Test by weighting total immediate and delayed recall scores. The domain information processing speed included the Stroop Color-Word Test Part I and II, the Concept Shifting Test Part A and B, and the Letter-Digit Substitution Test. Executive function was assessed by the Stroop Color-Word Test Part III and the Concept Shifting Test Part C. A brief description of the individual tests is provided below.

Raw test scores were transformed into z-scores. Standardized scores of the Stroop Color-Word Test and Concept Shifting Test were inverted so that higher scores indicated better cognitive performance. Thereafter, domain-specific scores were calculated as the standardized average of the z-scores from (sub)tests within that domain (e.g., memory function= z-score of (z-score_immediate recall_+ z-score_delayed recall_/ 2)). The standardized average of these domain scores was then considered a measure of overall cognitive performance (i.e., overall cognitive performance=z-score of (memory function+ information processing speed + executive function / 3)).

*Description of the individual cognitive tests used in the present study*

*Verbal Learning Test.^4^* Fifteen unrelated, monosyllabic, words were presented on a computer screen in five subsequent trials. After each trial, participants were instructed to recall as many words as possible in any order. Twenty minutes after the last trial, participants were asked again to reproduce the words. Outcomes recorded included the total number of words correctly recalled over the five trials (total immediate recall) and the number of correctly recalled words during delayed recall (delayed recall).

*Stroop Color-Word Test.^5^* In this test, which consisted of three parts, participants were firstly asked to read aloud color names (i.e., red, blue, yellow, and green) that were printed in black ink (Part I). Secondly, they were instructed to name solid color patches (Part II). Finally, participants had to name the ink color of color names that were printed in an incongruent color (e.g., participants were asked to say red when the word yellow was printed in red) (Part III). The time needed to complete Part III was adjusted for the average time needed to complete Part I and II.

*Concept Shifting Test.^6^* This test, a modification of the Trailing Making Test, consisted of four subtasks. During each subtask, participants were shown 16 small circles aligned along a larger imaginary circle. The small circles contained (a combination of) digits, letters, or were empty. Participants were instructed to cross-out as quickly as possible the digits in ascending order (Part A), the letters in alphabetic order (Part B), and the letters and digits in alternating order (Part C). Thereafter, participants were asked to cross-out empty circles in a clockwise fashion in two consecutive trials (Part 0). In this way, test results could be accounted for basic motor speed. The time needed to complete subtasks A and B was adjusted for the average time needed to complete Part 0, the time needed to completed Part C for the average time of Part A and B.

*Letter-Digit Substitution Test.^7^* Participants were requested to match digits to letters according to a given key. This key included the numbers 1 to 9, each paired with a different letter. The outcome of interest was the number of correct substitutions within 90 seconds.

**Supplemental Table 1. Clinical characteristics of study population and individuals excluded from the analyses due to missing values.**

| **Characteristics** | | Included study group  (N=6349) | | Excluded due to missing values  (N=2838) | Missing due to excluded values | | p |  |
| --- | --- | --- | --- | --- | --- | --- | --- | --- |
| **Demographic characteristics** | |  | |  |  | |  |  |
| Age (y) | | 59.35 ± 8.66 | | 59.97 ± 8.96 | 0 | | 0.002 |  |
| Female, No. (%) | | 3213 (50.6) | | 1403 (49.4) | 0 | | 0.300 |  |
| Educational level | |  | |  |  | |  |  |
| Low | | 2024 (31.9) | | 981 (36.2) | 126 | | <0.001 |  |
| Middle | | 1769 (27.9) | | 730 (26.9) |  | |  |  |
| High | | 2556 (40.3) | | 1001 (36.9) |  | |  |  |
| **Cardiovascular risk factors** | |  | |  |  | |  |  |
| Glucose metabolism status | |  | |  |  | |  |  |
| Normal glucose metabolism | | 4015 (63.2) | | 1732 (61.0) | 0 | | 0.183 |  |
| Prediabetes | | 949 (14.9) | | 433 (15.3) |  | |  |  |
| Type 2 diabetes | | 1350 (21.3) | | 655 (23.1) |  | |  |  |
| Type 1 and other type of diabetes | | 35 (0.6) | | 18 (0.6) |  | |  |  |
| Waist circumference (cm) | | 94.64 ± 13.48 | | 95.67 ± 13.89 | 5 | | <0.001 |  |
| Total/HDL cholesterol ratio | | 3.63 ± 1.19 | | 3.58 ± 1.16 | 6 | | 0.109 |  |
| Systolic blood pressure (mm Hg) | | 133.21 ± 17.89 | | 132.99 ± 18.07 | 4 | | 0.586 |  |
| **Medications** | |  | |  |  | |  |  |
| Use of lipid-modifying medication (yes versus no) | | 1825 (28.7) | | 883 (31.3) | 13 | | 0.015 |  |
| Use of blood pressure medications (yes versus no) | | 2218 (34.9) | | 1082 (38.3) | 12 | | 0.002 |  |
| **Lifestyle factors** | |  | |  |  | |  |  |
| Alcohol consumption | |  | |  |  | |  |  |
| None | 1132 (17.8) | 560 (20.3) | | | 75 | <0.001 |  |  |
| Low (women<=7, men<=14) | 3734 (58.8) | 1650 (59.7) | | |  |  |  |  |
| High (women>7, men>14) | 1483 (23.4) | 553 (20.0) | | |  |  |  |  |
| Smoking status | |  | |  |  | |  |  |
| Never | | 2406 (37.9) | | 1097 (39.6) | 71 | | 0.010 |  |
| Former | | 3136 (49.4) | | 1276 (46.1) |  | |  |  |
| Current | | 807 (12.7) | | 394 (14.2) |  | |  |  |
| **Heart rate variability** | |  | |  |  | |  |  |
| **Time Domain** | |  | |  |  | |  |  |
| SDNN, ms | | 142.08 ± 74.70 | | 145.57 ± 76.11 | 2294* | | 0.493 | |
| SDANN, ms | | 134.16 ± 96.18 | | 139.42 ± 104.10 |  | | 0.520 |  |
| RMSSD, ms | | 43.51 ± 67.99 | | 49.79 ± 78.63 |  | | <0.001 |  |
| SDNN index, ms | | 79.06 ± 115.36 | | 90.70 ± 136.12 |  | | 0.530 |  |
| pNN50, % | | 6.75 (2.90–14.05) | | 7.74 (3.54-15.98) |  | | 0.482 |  |
| **Frequency Domain** | |  | |  |  | |  |  |
| TP, ms^2^ | | 13,067 ± 7,439 | | 13,617 ± 7,435 |  | | 0.782 |  |
| ULF, ms^2^ | | 11,182 ± 6,653 | | 11,656 ± 6,649 |  | | 0.045 |  |
| VLF, ms^2^ | | 1,283 ± 878 | | 1,323 ± 845 |  | | <0.001 |  |
| LF, ms^2^ | | 471 ± 390 | | 501 ± 392 |  | | <0.001 |  |
| HF, ms^2^ | | 92.82 (51.58– 169.37) | | 105.44 (60.24– 192.70) |  | | <0.001 |  |
| **Cognitive Performance** | |  | |  |  | |  |  |
| Memory Function | | 0.04 ± 0.94 | | -0.10 ± 0.98 | 402 | | <0.001 |  |
| Information Processing Speed | | 0.03 ± 0.77 | | -0.05 ± 0.81 | 463 | | 0.077 |  |
| Executive function | | 0.03 ± 0.80 | | -0.05 ± 0.83 | 498 | | <0.001 |  |

Data are presented as mean ± standard deviation, median [interquartile range] or number (%). SD: standard deviation; HDL: high-density lipoprotein; *same population number for SDANN, RMSSD, SDNN index, pNN50, TP, ULF, VLF, LF, and HF.

**Supplemental Table 2. Associations of composite HRV measures with global cognitive performance, memory function, executive function and information processing speed.**

| N= 6,349  HRV measure |  | Global cognitive performance | | Memory function,  per SD | | Executive function,  per SD | | Information processing speed, per SD | |  |
| --- | --- | --- | --- | --- | --- | --- | --- | --- | --- | --- |
|  | Models | stβ (95%CI) | p | stβ (95%CI) | p | stβ (95%CI) | p | stβ (95%CI) | p |  |
| Time domain composite z-score, per SD † | 1 | **-0.05 (-0.033 to -0.065)** | **<0.001** | **-0.07 (-0.049 to- 0.096)** | **<0.001** | **-0.04 (-0.021 to -0.061)** | **<0.001** | **-0.03 (-0.014 to -0.053)** | **<0.001** | |
|  | 2 | -0.01 (0.002 to -0.024) | 0.074 | **-0.04 (-0.023 to -0.063)** | **<0.001** | -0.003 (0.015 to -0.020) | 0.763 | 0.01 (0.026 to -0.006) | 0.224 | |
|  | 3 | -0.01 (0.002 to -0.023) | 0.113 | **-0.04 (-0.021 to -0.061)** | **<0.001** | -0.001 (0.019 to -0.019) | 0.876 | 0.01 (0.027 to -0.005) | 0.171 | |
| Frequency domain composite z-score, per SD ‡ | 1 | **-0.10 (-0.087 to -0.119)** | **<0.001** | **-0.09 (-0.065 to -0.111)** | **<0.001** | **-0.11 (-0.093 to -0.132)** | **<0.001** | **-0.11 (-0.090 to -0.128)** | **<0.001** | |
|  | 2 | **-0.02 (-0.004 to -0.031)** | **0.013** | **-0.03 (-0.004 to -0.046)** | **0.020** | **-0.02 (-0.002 to -0.039)** | **0.029** | -0.01 (0.011 to -0.023) | 0.470 | |
|  | 3 | **-0.01 (-0.0001 to -0.028)** | **0.048** | **-0.02 (-0.002 to -0.044)** | **0.036** | -0.02 (0.001 to -0.036) | 0.060 | -0.001 (0.016 to -0.018) | 0.928 | |

Standardized regression coefficients (stβ) represent the differences in global cognitive performance, memory function, executive function and information processing speed in SD, for every 1 standard deviation (SD) lower HRV.

Variables entered in models: Model 1: crude; Model 2: adjusted for age, sex, glucose metabolism status, educational level; Model 3: additionally adjusted for waist circumference, alcohol consumption status, smoking status, total cholesterol-to-HDL cholesterol ratio, use of lipid-modifying medication, use of anti-hypertensive medication and office systolic blood pressure.

Bold denotes p<0.05.

† Time domain z-score combines SDNN, SDANN, RMSSD, SDNN index, and pNN50

‡ Frequency domain z-score combines TP, ULF, VLF, LF, and HF.

Stβ: standardized beta: CI: confidence interval; SD: standard deviation.

**Supplemental Table 3. Associations of individual time domain HRV measures with memory function, executive function and information processing speed.**

| N= 6,349 | |  | Memory function,  per SD | | Executive function,  per SD | | | Information processing speed, per SD | | |
| --- | --- | --- | --- | --- | --- | --- | --- | --- | --- | --- |
| Time domain | | Models | stβ (95%CI) | p | stβ (95%CI) | p | stβ (95%CI) | | p |  |
|  | SDNN, per SD | 1 | **-0.04 (-0.014 to -0.061)** | **0.002** | **0.04 (0.025 to 0.064)** | **<0.001** | **-0.04 (-0.017 to -0.055)** | | **<0.001** |  |
|  |  | 2 | -0.02 (0.005 to -0.035) | 0.143 | 0.01 (-0.010 to 0.025) | 0.393 | -0.000 (0.016 to -0.016) | | 0.958 |  |
|  |  | 3 | -0.01 (0.007 to -0.033) | 0.169 | 0.01 (-0.012 to 0.024) | 0.501 | 0.002 (0.018 to -0.014) | | 0.802 |  |
|  | SDANN, per SD | 1 | **-0.04 (-0.020 to -0.066)** | **<0.001** | **0.02 (0.002 to 0.041)** | **0.033** | **-0.02 (-0.003 to -0.041)** | | **0.026** |  |
|  |  | 2 | **-0.03 (-0.006 to -0.046)** | **0.010** | 0.000 (-0.017 to 0.018) | 0.993 | 0.002 (0.018 to -0.014) | | 0.778 |  |
|  |  | 3 | **-0.03 (-0.005 to -0.045)** | **0.016** | -0.001 (-0.019 to 0.016) | 0.884 | 0.004 (0.020 to -0.012) | | 0.606 |  |
|  | RMSSD, per SD | 1 | **-0.06 (-0.035 to -0.081)** | **<0.001** | **0.03 (0.006 to 0.045)** | **0.012** | -0.02 (0.005 to -0.034) | | 0.137 |  |
|  |  | 2 | **-0.03 (-0.012 to -0.052)** | **0.002** | -0.002 (-0.019 to 0.016) | 0.842 | **0.02 (0.034 to 0.002)** | | **0.025** |  |
|  |  | 3 | **-0.03 (-0.013 to -0.053)** | **0.001** | -0.001 (-0.019 to 0.016) | 0.882 | **0.02 (0.033 to 0.001)** | | **0.038** |  |
|  | SDNN index, per SD | 1 | **-0.07 (-0.050 to -0.096)** | **<0.001** | **0.05 (0.026 to 0.065)** | **<0.001** | **-0.04 (-0.017 to -0.055)** | | **<0.001** |  |
|  |  | 2 | **-0.04 (-0.024 to -0.064)** | **<0.001** | 0.01 (-0.011 to 0.024) | 0.455 | 0.01 (0.023 to -0.009) | | 0.360 |  |
|  |  | 3 | **-0.04 (-0.022 to -0.062)** | **<0.001** | 0.01 (-0.012 to 0.023) | 0.551 | 0.01 (0.025 to -0.007) | | 0.284 |  |
|  | pNN50, per SD | 1 | **-0.06 (-0.036 to -0.083)** | **<0.001** | 0.02 (-0.003 to 0.037) | 0.089 | -0.01 (0.005 to -0.033) | | 0.157 |  |
|  |  | 2 | **-0.04 (-0.021 to -0.061)** | **<0.001** | -0.002 (-0.020 to 0.015) | 0.781 | 0.01 (0.025 to -0.007) | | 0.250 |  |
|  |  | 3 | **-0.04 (-0.020 to -0.060)** | **<0.001** | -0.003 (-0.021 to 0.014) | 0.697 | 0.01 (0.025 to -0.006) | | 0.235 |  |

Standardized regression coefficients (stβ) represent the differences in memory function, executive function and information processing speed in SD, for every 1 standard deviation (SD) lower time domain HRV individual measures.

Variables entered in models: Model 1: crude; Model 2: adjusted for age, sex, glucose metabolism status, educational level; Model 3: additionally adjusted for waist circumference, alcohol consumption status, smoking status, total cholesterol-to-HDL cholesterol ratio, use of lipid-modifying medication, use of anti-hypertensive medication and office systolic blood pressure.

Bold denotes p<0.05.

Abstβ: standardized beta; CI: confidence interval; SD: standard deviation.

**Supplemental Table 4. Associations of individual frequency domain HRV measures with memory function, executive function and information processing speed.**

| N= 6,349 |  | Memory function,  per SD | | | Executive function,  per SD | | | Information processing speed, per SD | |
| --- | --- | --- | --- | --- | --- | --- | --- | --- | --- |
| Frequency domain | Models | stβ (95%CI) | p | stβ (95%CI) | | p | stβ (95%CI) | | p |
| TP, per SD | 1 | **-0.07 (-0.050 to -0.096)** | **<0.001** | **-0.09 (-0.066 to -0.106)** | | **<0.001** | **-0.09 (-0.068 to -0.106)** | | **<0.001** |
|  | 2 | **-0.02 (-0.002 to -0.043)** | **0.034** | -0.02 (0.002 to -0.034) | | 0.074 | **-0.02 (-0.001 to -0.034)** | | **0.039** |
|  | 3 | **-0.02 (-0.001 to -0.043)** | **0.038** | -0.02 (0.002 to -0.035) | | 0.114 | -0.02 (0.002 to -0.031) | | 0.084 |
| ULF, per SD | 1 | **-0.07 (-0.046 to -0.092)** | **<0.001** | **-0.07 (-0.053 to -0.092)** | | **<0.001** | **-0.09 (-0.068 to -0.106)** | | **<0.001** |
|  | 2 | -0.02 (-0.000 to -0.041) | 0.051 | -0.01 (0.005 to -0.031) | | 0.169 | **-0.02 (-0.002 to -0.035)** | | **0.027** |
|  | 3 | **-0.02 (-0.000 to -0.042)** | **0.049** | -0.01 (0.005 to -0.031) | | 0.159 | **-0.02 (-0.000 to -0.033)** | | **0.044** |
| VLF, per SD | 1 | **-0.06 (-0.032 to -0.078)** | **<0.001** | **-0.11 (-0.095 to -0.134)** | | **<0.001** | **-0.09 (-0.069 to -0.107)** | | **<0.001** |
|  | 2 | **-0.03 (-0.006 to -0.046)** | **0.011** | **-0.03 (-0.016 to -0.053)** | | **<0.001** | -0.01 (0.011 to -0.023) | | 0.482 |
|  | 3 | **-0.02 (-0.002 to -0.045)** | **0.031** | **-0.03 (-0.011 to -0.049)** | | **0.002** | 0.002 (0.023 to -0.010) | | 0.842 |
| LF, per SD | 1 | **-0.07 (-0.050 to -0.097)** | **<0.001** | **-0.12 (-0.056 to -0.095)** | | **<0.001** | **-0.11 (-0.090 to -0.128)** | | **<0.001** |
|  | 2 | -0.02 (0.004 to -0.039) | 0.108 | **-0.02 (-0.005 to -0.042)** | | **0.014** | -0.001 (0.016 to -0.018) | | 0.919 |
|  | 3 | -0.01 (0.008 to -0.034) | 0.236 | **-0.02 (-0.000 to -0.038)** | | **0.048** | 0.01 (0.024 to -0.011) | | 0.453 |
| HF, per SD | 1 | **-0.08 (-0.057 to -0.103)** | **<0.001** | **-0.05 (-0.032 to -0.071)** | | **0.001** | **-0.05 (-0.035 to -0.073)** | | **<0.001** |
|  | 2 | -0.01 (0.010 to -0.030) | 0.333 | 0.01 (0.023 to -0.012) | | 0.403 | **0.02 (0.034 to 0.002)** | | **0.028** |
|  | 3 | -0.01 (0.011 to -0.029) | 0.395 | 0.01 (0.025 to -0.010) | | 0.428 | **0.02 (0.035 to 0.003)** | | **0.018** |

Standardized regression coefficients (stβ) represent the differences in memory function, executive function and information processing speed in SD, for every 1 standard deviation (SD) lower frequency domain HRV individual measures.

Variables entered in models: Model 1: crude; Model 2: adjusted for age, sex, glucose metabolism status, educational level; Model 3: additionally adjusted for waist circumference, alcohol consumption status, smoking status, total cholesterol-to-HDL cholesterol ratio, use of lipid-modifying medication, use of anti-hypertensive medication and office systolic blood pressure.

Bold denotes p<0.05.

Stβ: standardized beta; CI: confidence interval; SD: standard deviation.

**Supplemental Table 5. Associations of composite time and frequency domains HRV with memory function, executive function and information processing speed additionally adjusted for kidney variables (estimated glomerular filtration; albuminuria), depressive symptoms (assessed with the Mini International Neuropsychiatric Interview) and use of anti-depressive medication, anxiolytic medication, sleep-modifying medication, physical activity and Dutch healthy diet score.**

|  |  | Global cognitive performance, per SD | | | Memory function,  per SD | | Executive function,  per SD | | | Information processing speed, per SD | |
| --- | --- | --- | --- | --- | --- | --- | --- | --- | --- | --- | --- |
|  | Models | stβ (95%CI) | p | stβ (95%CI) | | p | stβ (95%CI) | p | stβ (95%CI) | | p |
| Time domain composite z-score, per SD † | 4a | -0.01 (0.002 to -0.023) | 0.107 | **-0.04 (-0.021 to -0.061)** | | **<0.001** | -0.00 (0.016 to -0.019) | 0.874 | 0.01 (0.027 to -0.005) | | 0.185 |
|  | 4b | -0.01 (0.003 to -0.023) | 0.128 | **-0.04 (-0.022 to -0.063)** | | **<0.001** | -0.00 (0.017 to -0.018) | 0.963 | 0.01 (0.027 to -0.004) | | 0.159 |
|  | 4c | -0.01 (0.003 to -0.023) | 0.127 | **-0.04 (-0.021 to -0.061)** | | **<0.001** | -0.001 (0.017 to -0.019) | 0.909 | 0.01 (0.027 to -0.004) | | 0.158 |
|  | 4d | -0.01 (0.002 to -0.024) | 0.108 | **-0.04 (-0.021 to -0.061)** | | **<0.001** | -0.002 (0.016 to -0.019) | 0.863 | 0.01 (0.027 to -0.005) | | 0.180 |
|  | 3e* | -0.00 (0.013 to -0.016) | 0.864 | -0.02 (0.007 to -0.040) | | 0.162 | 0.00 (0.024 to -0.016) | 0.703 | 0.01 (0.027 to -0.009) | | 0.349 |
|  | 4e | -0.002 (0.012 to -0.017) | 0.743 | -0.02 (0.006 to -0.041) | | 0.137 | 0.004 (0.024 to -0.017) | 0.727 | 0.01 (0.025 to -0.012) | | 0.481 |
| Frequency domain composite z-score, per SD ‡ | 4a | -0.01 (-0.000 to -0.027) | 0.057 | **-0.02 (-0.000 to -0.043)** | | **0.047** | -0.02 (0.001 to -0.036) | 0.064 | -0.00 (0.017 to -0.017) | | 0.981 |
|  | 4b | -0.01 (0.002 to -0.025) | 0.093 | **-0.02 (-0.003 to -0.046)** | | **0.022** | -0.02 (0.004 to -0.033) | 0.117 | 0.001 (0.018 to -0.016) | | 0.882 |
|  | 4c | -0.01 (-0.000 to -0.027) | 0.056 | **-0.02 (-0.001 to -0.044)** | | **0.040** | -0.02 (0.001 to -0.036) | 0.067 | -0.00 (0.017 to -0.017) | | 0.968 |
|  | 4d | **-0.01 (-0.000 to -0.027)** | **0.053** | **-0.02 (-0.001 to -0.044)** | | **0.037** | -0.02 (0.001 to -0.036) | 0.065 | -0.00 (0.017 to -0.017) | | 0.968 |
|  | 3e* | -0.01 (0.004 to -0.026) | 0.163 | -0.02 (0.002 to -0.047) | | 0.070 | -0.02 (0.006 to -0.036) | 0.159 | 0.00 (0.023 to -0.015) | | 0.647 |
|  | 4e | -0.01 (0.003 to -0.028) | 0.110 | -0.02 (-0.000 to -0.048) | | 0.054 | -0.02 (0.005 to -0.037) | 0.145 | 0.002 (0.021 to -0.018) | | 0.870 |

Standardized regression coefficients (stβ) represent the differences in global cognitive performance, memory function, executive function and information processing speed in SD, for every 1 standard deviation (SD) lower HRV.

Variables entered in models: crude + adjusted for age, sex, glucose metabolism status, educational level, waist circumference, alcohol consumption status, smoking status, total cholesterol-to-HDL cholesterol ratio, use of lipid-modifying medication, use of anti-hypertensive medication, office systolic blood pressure :

Model 4a + eGFR (n=6345);

Model 4b + Mini international neuropsychiatric interview and use of anti-depressive medication (n=6314);

Model 4c + Sleep-modifying medication (n=6348);

Model 4d + Anxiolytic medication (n=6348);

Model 3e*

Model 4e + Physical activity and Dutch healthy diet score (n=4895);

Bold denotes P<0.05.

† Time domain z-score combines SDNN, SDANN, RMSSD, SDNN index and pNN50.

‡ Frequency domain z-score combines TP, ULF, VLF, LF, and HF.

Stβ: standardized beta; CI: confidence interval; SD: standard deviation; eGFR: estimated glomerular filtration.

**Supplemental Table 6. Associations of composite time and frequency domains HRV with memory function, executive function and information processing speed after exclusion of individuals that take antihypertensive medications.**

| N= 6,357 |  | Global cognitive performance, per SD | | Memory function,  per SD | | Executive function,  per SD | | Information processing speed, per SD | | | |
| --- | --- | --- | --- | --- | --- | --- | --- | --- | --- | --- | --- |
|  | Models | stβ (95%CI) | p | stβ (95%CI) | p | stβ (95%CI) | p | stβ (95%CI) | p | |  |
| Time domain composite z-score, per SD † | 1 | **-0.05 (-0.028 to -0.061)** | **<0.001** | **-0.07 (-0.045 to -0.091)** | **<0.001** | **-0.04 (-0.017 to -0.057)** | **<0.001** | **-0.03 (-0.010 to -0.048)** | **0.003** |  |  |
|  | 2 | -0.01 (0.002 to -0.024) | 0.095 | **-0.04 (-0.022 to -0.062)** | **<0.001** | -0.001 (0.016 to -0.019) | 0.896 | 0.01 (0.026 to -0.005) | 0.192 |  |  |
|  | 3 | -0.01 (0.003 to -0.023) | 0.127 | **-0.04 (-0.021 to -0.061)** | **<0.001** | -0.000 (0.017 to -0.018) | 0.982 | 0.01 (0.027 to -0.005) | 0.162 |  |  |
| Frequency domain composite z-score, per SD ‡ | 1 | **-0.08 (-0.061 to -0.093)** | **<0.001** | **-0.06 (-0.039 to -0.086)** | **<0.001** | **-0.09 (-0.068 to -0.107)** | **<0.001** | **-0.08 (-0.062 to -0.100)** | **<0.001** |  |  |
|  | 2 | -0.01 (0.0003 to -0.026) | 0.056 | **-0.02 (-0.001 to -0.042)** | **0.041** | -0.01 (0.004 to -0.032) | 0.118 | -0.003 (0.013 to -0.019) | 0.709 |  |  |
|  | 3 | -0.01 (0.004 to -0.023) | 0.155 | -0.02 (0.002 to -0.040) | 0.070 | -0.01 (0.007 to -0.030) | 0.213 | 0.002 (0.018 to -0.015) | 0.840 |  |  |

Standardized regression coefficients (stβ) represent the differences in global cognitive performance, memory function, executive function and information processing speed in SD, for every 1 standard deviation (SD) lower HRV.

Variables entered in models: Model 1: crude; Model 2: adjusted for age, sex, glucose metabolism status, educational level; Model 3: additionally adjusted for waist circumference, alcohol consumption status, smoking status, total cholesterol-to-HDL cholesterol ratio, use of lipid-modifying medication and office systolic blood pressure.

Bold denotes p<0.05.

† Time domain z-score combines SDNN, SDANN, RMSSD, SDNN index and pNN50

‡ Frequency domain z-score combines TP, ULF, VLF, LF, and HF.

Stβ: standardized beta; CI: confidence interval; SD: standard deviation.

**Supplemental Table 7. Associations of composite time and frequency domains HRV with memory function, executive function and information processing speed after replacement of waist circumference with body-mass index (BMI),** **office systolic blood pressure with office diastolic blood pressure, educational level with income, glucose metabolism status with fasting plasma glucose, 2-h post load glucose and HbA1c.**

|  |  | Global cognitive performance, per SD | | Memory function,  per SD | | Executive function,  per SD | | Information processing speed, per SD | |
| --- | --- | --- | --- | --- | --- | --- | --- | --- | --- |
|  | Models | stβ (95%CI) | p | stβ (95%CI) | p | stβ (95%CI) | p | stβ (95%CI) | p |
| Time domain composite z-score, per SD † | 5a | -0.01 (0.003 to -0.023) | 0.115 | **-0.04 (-0.022 to -0.062)** | **<0.001** | -0.001 (0.016 to -0.019) | 0.891 | 0.01 (0.028 to -0.004) | 0.142 |
|  | 5b | -0.01 (0.002 to -0.024) | 0.106 | **-0.04 (-0.020 to -0.060)** | **<0.001** | -0.002 (0.016 to -0.019) | 0.841 | 0.01 (0.026 to -0.006) | 0.226 |
|  | 5c | -0.01 (0.001 to -0.029) | 0.062 | **-0.05 (-0.022 to -0.069)** | **<0.001** | -0.01 (0.014 to -0.026) | 0.562 | 0.01 (0.026 to -0.010) | 0.375 |
|  | 5d | -0.01 (0.002 to -0.024) | 0.085 | **-0.04 (-0.022 to -0.062)** | **<0.001** | -0.003 (0.015 to -0.020) | 0.771 | 0.01 (0.026 to -0.005) | 0.197 |
|  | 5e | -0.01 (0.004 to -0.022) | 0.182 | **-0.04 (-0.021 to -0.062)** | **<0.001** | -0.001 (0.017 to -0.019) | 0.921 | 0.02 (0.031 to -0.001) | 0.069 |
|  | 5f | -0.01 (0.001 to -0.025) | 0.068 | **-0.04 (-0.022 to -0.062)** | **<0.001** | -0.003 (0.014 to -0.021) | 0.717 | 0.01 (0.025 to -0.007) | 0.267 |
| Frequency domain composite z-score, per SD ‡ | 5a | -0.01 (-0.000 to -0.027) | 0.051 | **-0.02 (-0.002 to -0.045)** | **0.030** | -0.02 (0.001 to -0.036) | 0.065 | 0.000 (0.017 to -0.017) | 0.995 |
|  | 5b | **-0.01 (-0.001 to -0.028)** | **0.041** | -0.02 (-0.000 to -0.043) | 0.051 | **-0.02 (-0.000 to -0.038)** | **0.046** | 0.003 (0.014 to -0.020) | 0.742 |
|  | 5c | -0.02 (-0.000 to -0.032) | 0.055 | **-0.03 (-0.001 to -0.051)** | **0.041** | -0.02 (0.002 to -0.041) | 0.074 | -0.002 (0.017 to -0.021) | 0.860 |
|  | 5d | **-0.02 (-0.002 to -0.029)** | **0.028** | **-0.03 (-0.003 to -0.046)** | **0.024** | **-0.01 (-0.001 to -0.039)** | **0.036** | -0.002 (0.015 to -0.019) | 0.853 |
|  | 5e | -0.01 (0.002 to -0.026) | 0.089 | **-0.02 (-0.002 to -0.046)** | **0.031** | -0.02 (0.001 to -0.037) | 0.068 | 0.01 (0.022 to -0.012) | 0.557 |
|  | 5f | **-0.02 (-0.003 to -0.030)** | **0.018** | **-0.03 (-0.005 to -0.048)** | **0.015** | **-0.02 (-0.002 to -0.039)** | **0.032** | -0.003 (0.014 to -0.020) | 0.717 |

Standardized regression coefficients (stβ) represent the differences in global cognitive performance, memory function, executive function and information processing speed in SD, for every 1 standard deviation (SD) lower HRV.

Results are adjusted for age, sex, glucose metabolism status, educational level, waist circumference, alcohol consumption status, smoking status, total cholesterol-to-HDL cholesterol ratio, use of lipid-modifying medication, use of anti-hypertensive medication and office systolic blood pressure.

5a: Replacement of waist circumference with body-mass index (BMI). (n=6350)

5b: Replacement of office systolic blood pressure with office diastolic blood pressure. (n=6347)

5c: Replacement of educational level with income (n=4908)

5d: Replacement of glucose metabolism status with fasting plasma glucose. (n=6346)

5e: Replacement of glucose metabolism status with 2-h post load glucose. (n=6000)

5f: Replacement of glucose metabolism status with HbA1c. (n=6340)

Bold denotes P<0.05.

† Time domain z-score combines SDNN, SDANN, RMSSD, SDNN index and pNN50

‡ Frequency domain z-score combines TP, ULF, VLF, LF, and HF.

Abbreviations: stβ, standardized beta: CI, confidence interval; SD, standard deviation.

**Supplemental Table 8. p-values for interaction by sex, age and type 2 diabetes in the associations of HRV domains with memory function, executive function and information processing speed.**

|  |  | **Memory function** | **Executive function** | **Information processing speed** | |
| --- | --- | --- | --- | --- | --- |
|  |  | p | p | p | |
| **Time domain composite z-score, per SD †** | | | | | |
|  | | | | | |
| -Sex |  | 0.925 | 0.669 | | 0.445 |
| -Type 2 diabetes |  | 0.092 | 0.892 | | 0.255 |
| - Age |  | 0.100 | 0.996 | | 0.975 |
| **Frequency domain composite z-score, per SD‡** |  |  |  | |  |
|  |  |  |  | |  |
| -Sex |  | 0.682 | 0.236 | | 0.251 |
| -Type 2 diabetes |  | 0.443 | 0.433 | | 0.063 |
| -Age |  | 0.960 | 0.536 | | 0.436 |

Results are adjusted for age, sex, HbA1c, educational level, waist circumference, alcohol consumption status, smoking status, total cholesterol-to-HDL cholesterol ratio, use of lipid-modifying medication, use of anti-hypertensive medication and office systolic blood pressure.

Bold denotes p<0.05.

† Time domain z-score combines SDNN, SDANN, RMSSD, SDNN index, and pNN50

‡ Frequency domain z-score combines TP, ULF, VLF, LF, and HF.

SD: standard deviation.

**References**

1. Eaton JW, Bateman D, Hauberg S, et al. GNU Octave, https://www.gnu.org/software/octave/ (2020, accessed 21 October 2019).

2. Heart rate variability: standards of measurement, physiological interpretation and clinical use. Task Force of the European Society of Cardiology and the North American Society of Pacing and Electrophysiology. *Circulation* 1996; 93: 1043-1065.

3. Sassi R, Cerutti S, Lombardi F, et al. Advances in heart rate variability signal analysis: joint position statement by the e-Cardiology ESC Working Group and the European Heart Rhythm Association co-endorsed by the Asia Pacific Heart Rhythm Society. *Europace* 2015; 17: 1341-1353.

4. Van der Elst W, van Boxtel MP, van Breukelen GJ, et al. Rey's verbal learning test: normative data for 1855 healthy participants aged 24-81 years and the influence of age, sex, education, and mode of presentation. *J Int Neuropsychol Soc* 2005; 11: 290-302.

5. Van der Elst W, Van Boxtel MP, Van Breukelen GJ, et al. The Stroop color-word test: influence of age, sex, and education; and normative data for a large sample across the adult age range. *Assessment* 2006; 13: 62-79.

6. Van der Elst W, Van Boxtel MP, Van Breukelen GJ, et al. The Concept Shifting Test: adult normative data. *Psychol Assess* 2006; 18: 424-432.

7. van der Elst W, van Boxtel MP, van Breukelen GJ, et al. The Letter Digit Substitution Test: normative data for 1,858 healthy participants aged 24-81 from the Maastricht Aging Study (MAAS): influence of age, education, and sex. *J Clin Exp Neuropsychol* 2006; 28: 998-1009.
